# Diagnosis: Chronic Osteomyelitis

**Published:** 2014-03-27

**Authors:** Blair York, Jeon Cha, Alan Dao, Sam Gane, Igor Policinski, Mark Rahman

**Affiliations:** The Sydney Hand Unit, Sydney Hospital and Sydney Eye Hospital, Sydney, Australia

**Keywords:** chronic wounds, chronic infections, diagnosis of osteomyelitis, pathological changes in osteomyelitis, classification of osteomyelitis

## DESCRIPTION

**Figure 1:**
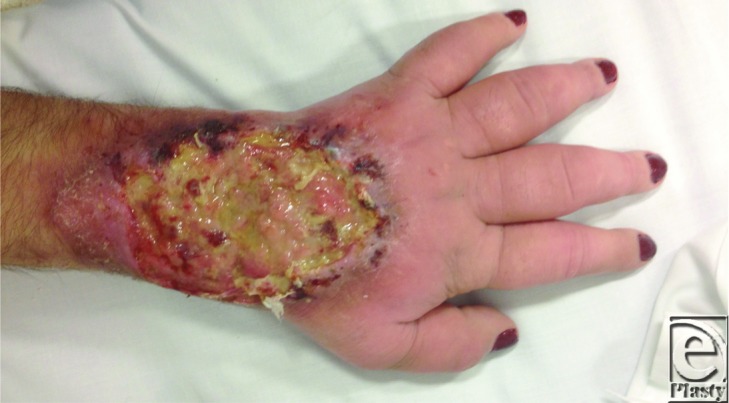
Longstanding, nonhealing, infected wound to the dorsum of left hand.

A 27-year-old woman presented with a chronic wound to the left forearm that had been previously treated at other medical institutions (Fig 1). On history, the wound had been present for more than 18 months with gradual worsening over this period of time.

## QUESTIONS

1. **What are the appropriate laboratory investigations for a patient with suspected chronic osteomyelitis?**
2. **What are the underlying pathological changes involved in osteomyelitis?**
3. **What imaging modalities are available to assist in the diagnosis and management of osteomyelitis?**
4. **What is the Cierny-Mader Classification for chronic osteomyelitis?**

## DISCUSSION

A thorough history and examination is required from any patient that presents with a chronic wound with suspected underlying skeletal involvement (Fig 1). The initial investigations are directed at defining and confirming the suspected pathology. These include histopathology of any involved soft and bony tissues (to exclude malignancy), microbiology to identify possible pathogens, inflammatory markers (C-reactive protein, erythrocyte sedimentation rate), and white cell counts. Other tests are conducted to determine if there are any reversible or irreversible host factors that may have exacerbated the condition or prevented normal healing (albumin/protein, hemoglobin, electrolytes, serology).^1^^,^^2^

Osteomyelitis is an inflammatory disorder of bone resulting from active infection that may lead to necrosis and destruction of both bone and associated soft tissue. It may develop from a number of causes including direct trauma resulting in inoculation, as a consequence to injury or infection to surrounding soft tissues, following bacteremia and secondary to conditions leading to vascular insufficiency. Acute osteomyelitis evolves over several days or weeks. If clinical signs persist for more than 10 days, bone necrosis may occur. Chronic osteomyelitis is typically defined by characteristic histopathological findings such as the persistence of microorganisms, low-grade inflammation, the presence of devitalized bone (sequestrum), new bone (involucrum) formed in response to the sequestra, fistulous tracts (cloacae), and soft tissue involvement.^1^^-^4

**Figure 2:**
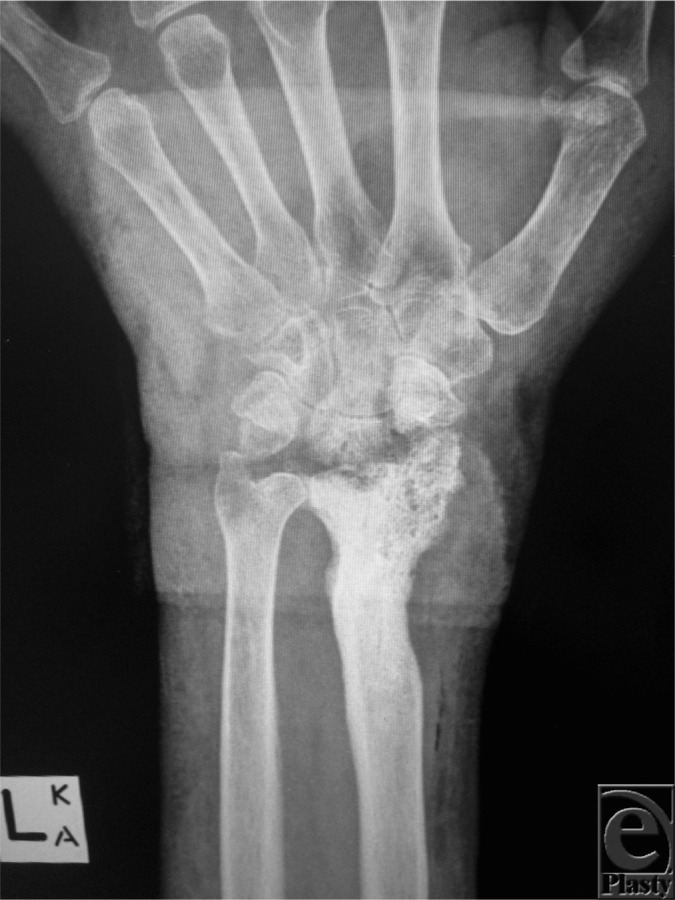
Plain radiograph of the patient's left forearm and wrist demonstrating underlying chronic osteomyelitis. There is evidence of osteolysis, sequestra, involucrum, and permeative changes to the distal radius and the radiocarpal joint.

Imaging modalities can be useful in confirming the diagnosis and also to further characterize the extent of osteomyelitis. Plain radiography is a useful first line modality to rule out other diagnoses and to monitor future progress. Typically, plain radiographs do not show evidence of osteomyelitis until 10 to 14 days after the onset of infection. Features that may be present include nonspecific periosteal reaction, osteolysis, a dense intramedullary cortical sequestrum, endosteal scalloping, or an involucrum (Fig 2). Computer tomography is useful to determine the extent of bony destruction or to guide biopsies and is the mainstay for surgical planning. Its role in diagnosis, however, is more limited compared with other modalities. Magnetic resonance imaging is useful in detecting osteomyelitis from 3 to 5 days after onset (with a specificity and sensitivity of >90%) in the absence of surgical hardware. It has the ability to delineate abscesses, sinus tracts, and soft tissue involvement in relation to bony involvement. Nuclear imaging can demonstrate evidence of osteomyelitis within a few days of onset. Triple phase Technetium-99 scans are able to detect regions of increased bone metabolism and is highly sensitive while leukocyte scintography is more specific for infection and can delineate areas of inflammation. They can be combined to improve the specificity and sensitivity of the diagnosis. Fluorodeoxyglucose positron emission tomography is the most sensitive and specific technique for diagnosing chronic osteomyelitis. It is not, however, routinely available or used for this purpose.^1^^,^^3^^,^^5^^,^^6^

The Cierny-Mader classification is widely used for adult chronic osteomyelitis. It is based on the osseous involvement and the physiologic status of the patient (host). Table 1 outlines this classification system as follows.^2^^,^^7^

Chronic osteomyelitis is a difficult entity to treat. Its diagnosis is based on clinical suspicion (history and clinical signs) and aided by laboratory and imaging studies. In severe chronic osteomyelitis, as in this case, intravenous antibiotics alone are unlikely to result in resolution. Source control with surgical debridement, long-term antibiotics, and definitive soft tissue coverage are needed for a successful outcome. Although the surgical options were discussed, the patient in this case self-discharged during the assessment period due to social circumstances and later sought treatment at another institute.

## Figures and Tables

**Table 1:** Cierny-Mader classification of chronic osteomyelitis^2^^,^^7^

Anatomic type	
Type I	Medullary—limited to the medullary canal
Type II	Superficial—infection is limited to the exterior to the bone and does not permeate the cortex
Type III	Permeative/Stable—infection penetrates cortex of bone but is axially stable
Type IV	Permeative/Unstable—infection throughout the bone in segmental fashion with axial instability
Host/Patient factors	
Type A	Normal physiological host
Type B	s—Systemic compromise
	l—Local compromise
	sl—Both systemic and local compromise
Type C	Treatment morbidity worse than present condition
